# Health indicator recording in UK primary care electronic health records: key implications for handling missing data

**DOI:** 10.2147/CLEP.S191437

**Published:** 2019-02-11

**Authors:** Irene Petersen, Catherine A Welch, Irwin Nazareth, Kate Walters, Louise Marston, Richard W Morris, James R Carpenter, Tim P Morris, Tra My Pham

**Affiliations:** 1 Department of Primary Care and Population Health, University College London, London NW3 2PF, UK, i.petersen@ucl.ac.uk; 2 Department of Clinical Epidemiology, Aarhus University, 8200 Aarhus N, Denmark, i.petersen@ucl.ac.uk; 3 Department of Health Sciences, University of Leicester, Leicester LE1 7RH, UK; 4 Department of Population Health Sciences, Bristol Medical School, University of Bristol, Bristol BS8 2PS, UK; 5 MRC Clinical Trials Unit at UCL, London WC1V 6LJ, UK; 6 Department of Medical Statistics, London School of Hygiene and Tropical Medicine, London WC1E 7HT, UK

**Keywords:** primary care, EHRs, recording, QOF, multiple imputation, statistics, epidemiology, research methods, data analysis

## Abstract

**Background:**

Clinical databases are increasingly used for health research; many of them capture information on common health indicators including height, weight, blood pressure, cholesterol level, smoking status, and alcohol consumption. However, these are often not recorded on a regular basis; missing data are ubiquitous. We described the recording of health indicators in UK primary care and evaluated key implications for handling missing data.

**Methods:**

We examined the recording of health indicators in The Health Improvement Network (THIN) UK primary care database over time, by demographic variables (age and sex) and chronic diseases (diabetes, myocardial infarction, and stroke). Using weight as an example, we fitted linear and logistic regression models to examine the associations of weight measurements and the probability of having weight recorded with individuals’ demographic characteristics and chronic diseases.

**Results:**

In total, 6,345,851 individuals aged 18–99 years contributed data to THIN between 2000 and 2015. Women aged 18–65 years were more likely than men of the same age to have health indicators recorded; this gap narrowed after age 65. About 60–80% of individuals had their height, weight, blood pressure, smoking status, and alcohol consumption recorded during the first year of registration. In the years following registration, these proportions fell to 10%–40%. Individuals with chronic diseases were more likely to have health indicators recorded, particularly after the introduction of a General Practitioner incentive scheme. Individuals’ demographic characteristics and chronic diseases were associated with both observed weight measurements and missingness in weight.

**Conclusion:**

Missing data in common health indicators will affect statistical analysis in health research studies. A single analysis of primary care data using the available information alone may be misleading. Multiple imputation of missing values accounting for demographic characteristics and disease status is recommended but should be considered and implemented carefully. Sensitivity analysis exploring alternative assumptions for missing data should also be evaluated.

## Introduction

Clinical and administrative health databases, such as disease registers, health insurance claim databases, and primary care electronic health record databases, have long been recognized as rich data sources for health research. There are several primary care databases in the UK, such as The Health Improvement Network (THIN),^1^,^2^ Clinical Practice Research Datalink,^3^ and QRESEARCH,^4^ which typically include several hundred geographically dispersed general practices with data collected since the early 1990s. These databases offer many opportunities for research using primary care data that would otherwise be prohibitively difficult and/or expensive to undertake. This includes research on populations that would be difficult to enroll in clinical trials or cohort studies, eg, patients with severe mental illness, pregnant women, children, and the very elderly. Primary care electronic health records have also proven to be very powerful tools for research into chronic diseases including diabetes, coronary heart disease, and stroke,^5^–^12^ which remain leading causes of the global disease burden.^13^

In tandem with appropriate design, research using electronic health records on chronic diseases often requires individual information on common health indicators such as height, weight, blood pressure, cholesterol level, as well as lifestyle factors including smoking status and alcohol consumption. These data are captured in UK primary care databases as part of the individuals’ routine consultations in primary care. However, because they are not always directly relevant to the clinical need behind a consultation, such data are not recorded on a regular basis as in cohort studies or clinical trials. Therefore, missing data are often an issue, and this raises significant challenges for statistical analysis and interpretation.^14^,^15^ A commonly used approach is to include only individuals with a complete record when analyzing these data (ie, a complete record analysis). However, the lack of any schedule for when data should be recorded means that a “complete record” is an undefined concept. In addition, a sufficient assumption for a complete record analysis to be valid is that the reason for data recording does not relate to any variables in the substantive analysis model (either missing or observed, also known as missing completely at random).^16^,^17^ However, this is rarely met in practice.^18^ More generally, using complete records to fit a substantive analysis model will be valid, if the probability of being a complete record is unrelated to the dependent variable given the covariates.^19^,^20^ Once again, this is unlikely to hold in practice.

In this study, we aimed to further understand how health indicators are recorded in the UK primary care setting, and if complete record analysis is a valid approach for dealing with missing data in primary care databases. Our objectives were to describe the recording of key health indicators in accordance with demographic variables (age and sex) and chronic diseases (diabetes, myocardial infarction, and stroke), as well as over time. In addition, we sought to assess the plausibility of the assumptions for how these data were missing (ie, missingness mechanisms). Specifically, we examined the associations of recorded values of a specific health indicator (weight) and the reason for data recording with individuals’ demographic characteristics and disease status.

## Methods

### Data source

We used data from THIN^1^ primary care database, one of the largest UK databases to provide longitudinal health records of individuals in primary care. We focused on data recorded from January 1, 2000 (or later, depending on when general practices met quality standards for data recording) to December 31, 2015. Two measures of data quality assurance at the general practice level have been derived: the acceptable mortality recording (AMR)^21^ and acceptable computer usage (ACU)^22^ dates. AMR defines the date when general practices recorded the date of death to an expected standard. ACU defines the date when general practices were generally using their computer system instead of paper-based records to document patient consultations. THIN has been shown to be broadly a representative of the UK population in terms of demographics and prevalence of major conditions.^2^

THIN contains individual-level information such as year of birth, date of first registration with the general practice, date of death, and date of transfer out of the practice. In addition, the database holds longitudinal information on patient consultations and medications prescribed in primary care. Diagnoses and symptoms are recorded by practice staff (general practitioners [GPs], nurses, and administrative staff) using Read codes,^23^,^24^ a hierarchical coding system. THIN also captures additional health data on height, weight, blood pressure, cholesterol level, smoking status, and alcohol consumption. These measurements are typically (but not always) recorded soon after the individual is registered with the general practice, and thereafter when relevant for routine clinical care.

The Quality and Outcomes Framework (QOF)^25^ was introduced in UK primary care in 2004. Under this scheme, GPs receive remuneration based on quality targets and they have to record data, eg, health measurements, in order to meet these targets. Since QOF began, many individuals with chronic conditions/illnesses have had their health indicator measurements recorded on a regular basis.^26^,^27^

### Study population

Individuals aged 18–99 years and permanently registered with general practices contributing data to THIN were followed from the latest of the date of registration with the practice, date when the practice recorded data to the standard defined by the AMR or ACU (see section “Data Source”), or January 1, 2000; until the earliest of the date of death, date of transfer out of the practice, or December 31, 2015.

### Data analyses

We examined the recording of the following routine health indicators: height, weight, blood pressure, total cholesterol, smoking status, and alcohol consumption.

First, we examined the annual recording of the aforementioned health indicators if the individuals had at least one measurement recorded during each calendar year of follow-up. We calculated the annual recording rate per 100 person-years for men and women aged 18–99 years during the follow-up period.

Second, we identified three cohorts of individuals who were newly registered with general practices in THIN in 2000, 2005, and 2010, and examined the recording of health indicators in these cohorts. Individuals were 18–99 years old at registration. We examined whether these individuals had any health indicator measurements recorded and how long after registration these measurements were recorded. We also calculated the proportions of men and women with at least one measurement of each health indicator recorded by calendar year after registration. We were aware that the recording of health indicators in primary care may depend on whether the individual has a chronic disease. To illustrate this, we stratified the analyses on whether the individuals had a record indicative of diabetes, myocardial infarction, or stroke; these are conditions defined by the QOF scheme and are likely to be associated with increased recording of the aforementioned health indicators (ie, cardiovascular risk factors).^28^

We then fitted Kaplan–Meier “time-to-measurement” curves to estimate the cumulative probability of men and women in the 2010 registration cohort (chosen for illustrative purpose) having at least one record of each health indicator during their follow-up. We also calculated the *p*-percentile of time-to-measurement with 95% CI for both men and women in this registration cohort. This is the analysis time at which *p*% of the individuals have had the first measurement recorded and (1 – *p*)% have not; *p*=50 for height, weight, SBP, alcohol consumption; *p*=25 for total cholesterol; *p*=75 for smoking status.

Finally, we assessed the missing completely at random assumption for the incomplete health indicator data by exploring potential predictors of the health indicator measurements and the probability of having the health indicator recorded, using weight as an example. We used linear regression analysis to examine the association of the mean weight measurements in 2010 (in kg) with sex, 5-year age group (18–99 years old), social deprivation (in quintiles of the Townsend deprivation score),^29^ and indicators of chronic diseases (diabetes, myocardial infarction, and stroke) among individuals who were actively registered in THIN in 2010. We also used logistic regression analysis to examine the association of the probability of weight being recorded with sex, age group, social deprivation, and chronic diseases. For those with multiple weight measurements in 2010, the latest record was chosen.

All analyses were conducted in Stata 15.1.^30^

### Ethics approval

The data provider (IQVIA) obtained overall ethical approval for the use of THIN in scientific research from the South East Medical Research Ethics Committee (MREC/03/01/073) and this study was further approved by the THIN Scientific Review Committee.

## Results

In total, 6,345,851 individuals (3,070,711 [48%] men and 3,275,140 [52%] women) aged 18–99 years were registered with 642 general practices contributing data to THIN between January 1, 2000 and December 31, 2015. The median follow-up times were 6.3 years (first to third quartiles 3.0–11.7) for men and 6.2 years (first to third quartiles 2.9–11.9) for women.

**Figure 1:**
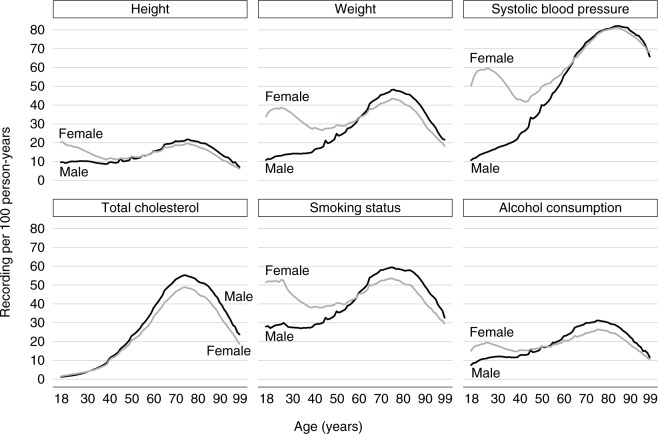
Number of records of each health indicator per 100 person-years by sex and age (in years).

The annual recording of health indicators varied with age and sex (Figure 1). The annual recording of height, weight, blood pressure, smoking status, and alcohol consumption was higher for women aged 18–65 years compared with men of the same age group. This gap was most marked at child-bearing ages. After age 65, there was little difference in the annual recording of height and SBP per 100 person-years between men and women; for other health indicators, the annual recording was slightly higher among men (Figure 1). In general, the annual recording fell as age increased >75 years. For total cholesterol, the annual recording was similar between men and women before age 50; recording increased from the age of 40 years for both men and women and peaked at age 75 (Figure 1).

**Figure 2:**
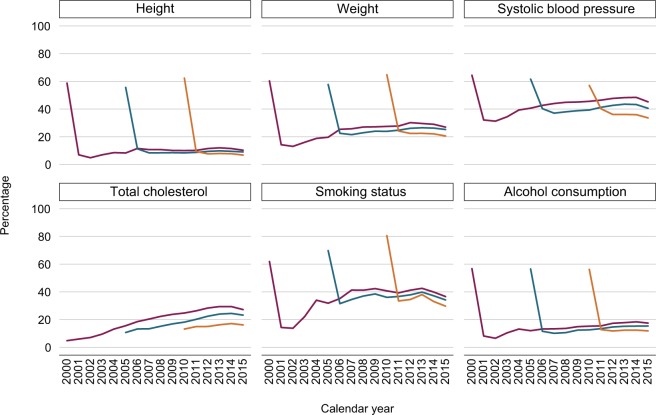
Percentage of individuals with a record of each health indicator in the 2000 (purple), 2005 (teal), and 2010 (orange) registration cohorts by calendar year. **Note:** The 2000, 2005, and 2010 registration cohorts included individuals who were newly registered with their general practices in 2000, 2005, and 2010, respectively.

In each of the three registration cohorts (2000, 2005, 2010), there were more women (52%–53%) who were registered than men (47%–48%; Table 1); the median age at registration in these cohorts was 34–35 years. Around 60% of individuals had a record of height, weight, SBP, and alcohol consumption in the first year after registration (Figure 2). In subsequent years, the proportion of individuals with a record of these health indicators dropped noticeably; eg, only 10%–20% had at least one weight measurement recorded (Figure 2). For smoking status, the number of individuals who had a record in the first year after registration increased in the more recent registration cohorts. In the 2010 registration cohort, 80% of individuals had a record of smoking status in the year after registration, while only 30%–40% of them had their smoking status recorded in subsequent years (Figure 2). The recording of total cholesterol differed from that of the other health indicators. Less than 10% of individuals who were newly registered in 2000 had a total cholesterol measurement during their first year after registration (Figure 2); this number almost doubled in the 2010 registration cohort. For all three registration cohorts, there was an increase in the proportion of individuals who had a total cholesterol measurement in the years following their registration with the general practices (Figure 2).

**Figure 3:**
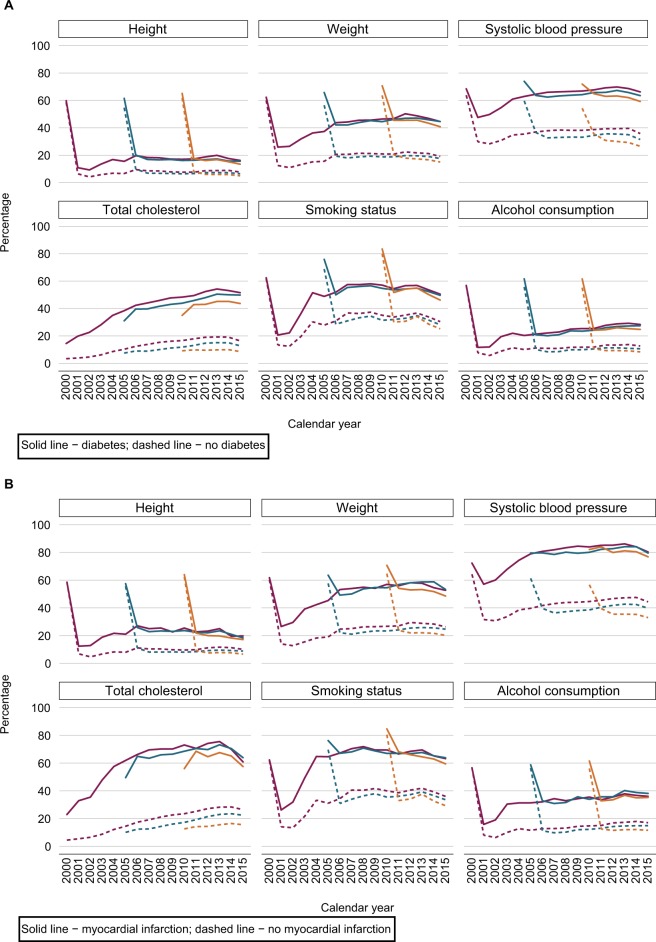

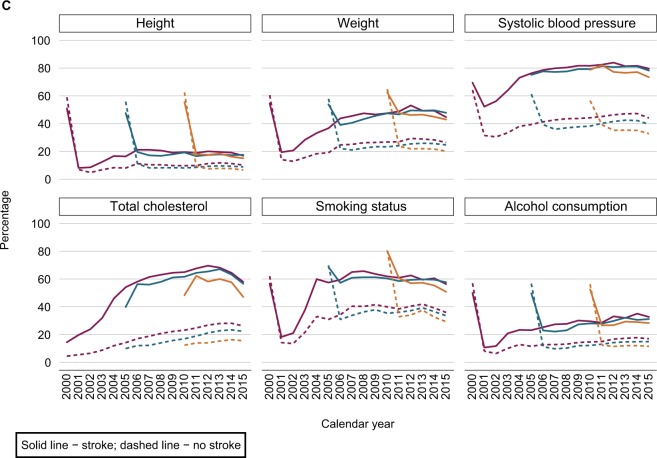
Percentage of individuals with a record of each health indicator in the 2000 (purple), 2005 (teal), and 2010 (orange) registration cohorts by calendar year and disease status. **Notes: (A)** Diabetes, **(B)** myocardial infarction, and **(C)** stroke. The 2000, 2005, and 2010 registration cohorts included individuals who were newly registered with their general practices in 2000, 2005, and 2010, respectively.

Recording of health indicators was improved after the introduction of QOF in 2004 (see section “Data source”). Figures 3A–C illustrate the completeness of the recording of height, weight, SBP, total cholesterol, smoking status, and alcohol consumption over time for the three registration cohorts, stratified by individuals with and without a diagnosis of diabetes, myocardial infarction, or stroke. These figures show that individuals with chronic diseases were much more likely to have their health indicators recorded compared with those who did not have the diseases.

**Figure 4:**
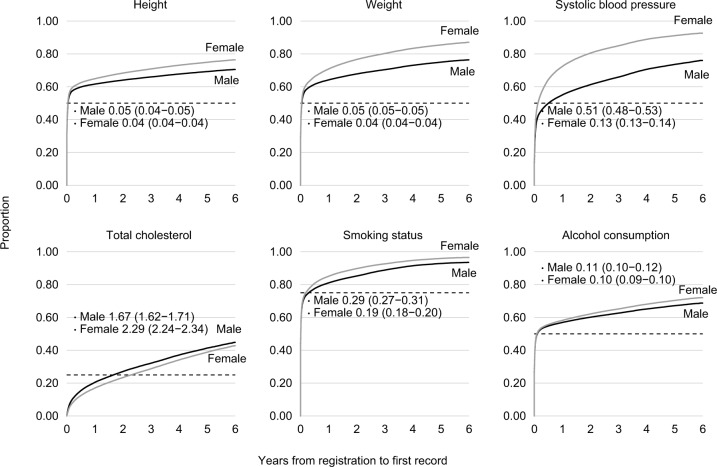
Time (in years) from practice registration to having the first record of each health indicator; and time (in years) at which 1) 50% of the individuals have had their first height, weight, SBP, or alcohol consumption record; 2) 25% of the individuals have had their first total cholesterol record; and 3) 75% of the individuals have had their first smoking status record.

For individuals in the 2010 registration cohort, the proportion of those who had a health indicator record was generally higher among women compared with men (Figure 4). Nearly all women had at least one measurement of weight and SBP and one record of smoking status during their time registered with the general practices (Figure 4). By contrast, men were less likely to have a record during their follow-up. One exception was total cholesterol for which the proportion of individuals who had a record was higher among men, but overall, only <50% of individuals had a record by the end of their follow-up (Figure 4). Women tended to have their first health indicator measurement recorded earlier than men. For example, 50% of women had their first record of SBP at 0.13 (95% CI 0.13–0.14) years after registration (ie, <2 months), whereas this was 0.51 (95% CI 0.49–0.53) years for men (ie, 6 months), indicating earlier recording of SBP for women (Figure 4).

In total, there were 3,583,437 individuals who were actively registered with general practices in THIN in 2010, of whom 1,105,741 (31%) had a weight measurement in 2010 and 2,477,696 (69%) did not. Table 2 describes adjusted associations of the mean weight measurements and the probability of having weight recorded with sex, age group, social deprivation, and indicators of chronic diseases. All demographic characteristics and disease indicators considered were predictive of both the observed weight measurement values and the probability of having a weight measurement recorded. This suggested that data on weight were not likely to be missing completely at random.^18^,^31^

## Discussion

In summary, our findings suggested that there were differences in the recording of health indicators by sex, age, and time since the individuals were first registered with their general practices. Likewise, we found that individuals with chronic conditions were more likely to have their health indicators recorded than those without, particularly after the introduction of QOF in 2004.

The recording of health indicators in general practices followed, to some extent, the consultation patterns by age and sex.^32^ In particular, younger women were more likely to consult their GPs than younger men. It seemed likely that for women, many weight and SBP measurements may have been taken in conjunction with their consultations for contraception and pregnancy. The New Patient Health Check scheme was introduced in UK primary care in 1995; although it is no longer a part of the general practice’s payment-for-performance, our results suggested that many general practices still offer these checks for their newly registered patients.

We found, similar to others, that the QOF scheme had a major impact on the recording of health indicators in patients with chronic diseases.^33^ Bhaskaran et al^15^ also observed similar recording patterns in the Clinical Practice Research Datalink^3^ primary care database, with more frequent weight recording in more recent years for patients with type 2 diabetes compared with those who did not have type 2 diabetes.

Unlike other health indicators, the pattern of total cholesterol recording was different, and fewer individuals had a measurement in the first year after registration. As part of the National Health Service (NHS) Health Check scheme, cholesterol screening is offered to individuals aged 40–74 years old who have not had a stroke, or do not already have heart disease, diabetes, or kidney disease; however, uptake of this service for the first quarter of 2011 was only around 50% in England.^34^ For patients who have a cardiovascular-related disease such as diabetes or myocardial infarction, they will have regular repeated cholesterol tests done as part of their routine clinical care. For those presenting with other cardiovascular risk factors such as obesity or raised blood pressure, they would also usually be offered a cholesterol test. This information would then be used to calculate a cardiovascular risk score. It would be unusual for individuals under the age of 40 years to be offered a cholesterol test, unless there is a good clinical reason for increased cardiovascular risk, eg, diabetes, a previous cardiovascular disease event, or a previous family history of hyperlipidemia. There was an increase in the recording of total cholesterol after 1999 when the prescription of statins, a lipid-modifying drug that helps lower cholesterol level, became more common.^35^ Patients prescribed with statins therefore tend to have their total cholesterol measured more frequently for monitoring cholesterol reduction. However, there is no evidence to suggest the benefit of statins in people who are >85 years old, and evidence for benefit in the 75–84 years age group is mixed. These are consistent with our findings that total cholesterol recording started to increase from the age of 40 years, peaked at age 75, and decreased thereafter.

Research based on electronic health records often involves the analysis of common health indicators. Missing data have proven to be a challenge in such research and, to handle missing data, various ad hoc approaches have been applied. Typically, these include a complete record analysis, using only individuals with complete information on all variables of interest in the analysis; the exclusion of variables with incomplete data from the analysis; or the creation of a separate category for missing values in the incomplete variables. The issue of bias and potentially incorrect conclusions from using these methods is well recognized.^18^,^36^–^38^ Using weight measurements recorded for individuals who were registered with general practices contributing data to THIN in 2010, we found that both the observed weight measurements and missingness in weight were associated with sex, age, social deprivation, and disease status. In an analysis where the outcome variable was disease status and covariates included sex, age, social deprivation alongside weight, the results from a complete record analysis involving weight in a given year would be susceptible to bias (see section “Introduction”). Complete record analysis can also substantially reduce the sample size and thereby the power of the studies if there is a large proportion of individuals who do not have the relevant data.

Multiple imputation of missing data, therefore, emerges as a potential alternative for handling missing data in large clinical databases.^14^,^37^,^39^,^40^ The standard implementation of multiple imputation is based on the assumption of data being missing at random where the reason for the missing values is not associated with the missing data, conditional on the observed data. Indeed, Marston et al^14^ examined the feasibility of multiple imputation for missing values in health indicators recorded in the first year after registration in THIN, and reported that the results were comparable with population surveys. Similarly, we found that the missing at random assumption was most plausible in the first year after registration, because data were mainly recorded for patient health monitoring afterward. However, the plausibility of this assumption can be enhanced by including in the imputation model indicators of disease status (such as diabetes, myocardial infarction, and stroke) that predict both missingness and the underlying missing values. The missing at random assumption may be less plausible for certain health indicators, eg, if individuals with high or low levels of the health indicators are monitored. While this cannot be verified purely through analysis of the observed data, we can use our knowledge of the clinical setting where data were recorded to understand why they were missing. When there are external data sources containing population information about the incomplete health indicators (eg, population censuses or surveys), such information can be utilized in a sensitivity analysis to explore potential departures from the missing at random assumption.^41^

Health research often uses data from a specific calendar date rather than the year of registration as the start of follow-up, eg, individuals are often followed from the time they turn 18 years of age or perhaps later in life for chronic diseases. The results of our study suggested that multiple imputation is an attractive and practical option for handling missing health indicator values in this setting, although care needs to be taken on correctly reflecting the structure of the substantive analysis model and accounting for nonlinear relationships.^42^ Additionally, the fact that many individuals may have had more than one record of height, weight, SBP, total cholesterol, smoking status, and alcohol consumption during follow-up suggested that an imputation strategy that exploits individual longitudinal trajectories might be preferred. Practical methods for longitudinal multiple imputation of repeated measurements of health indicators over time are increasingly available, such as the two-fold fully conditional specification algorithm,^43^–^45^ enabling a more efficient use of the full longitudinal records in analysis.

## Conclusion

For many health research studies using primary care electronic health records, missing data in key health indicators may be a major issue. The recording of common health indicators in primary care was found to vary by time after registration with the general practices, age, sex, and disease status. Multiple imputation that takes into account these factors is an attractive and practical option for handling missing data in such studies.

## Figures and Tables

**Table 1:** Number of individuals, median age at registration, and sex distribution among those who were newly registered with general practices in 2000, 2005, and 2010

Year of registration	Number of practices	Number of individuals	Median (Q1–Q3^a^) age at registration in years	Sex, n (%)	
				Male	Female
2000	635	180,871	35 (27–50)	86,179 (48)	94,692 (52)
2005	640	215,609	34 (26–48)	102,367 (47)	113,242 (53)
2010	607	195,491	34 (26–47)	91,970 (47)	103,521 (53)

**Note:**

a Q1, Q3: first and third quartiles, respectively.

**Table 2:** Associations of the mean weight measurements and the probability of having weight recorded with sex, age group, social deprivation, and indicators of chronic diseases among individuals who were actively registered in 2010

Variables	Differences in the mean weight measurements (n=1,104,221)			Differences in the probability of having weight recorded (n=3,583,437)		

	Difference in mean (kg)^a^	95% CI	*P*^b^	OR^c^	95% CI	*P*^b^

**Sex**			<0.001			<0.001
Men	Base level			1.00	1.55–1.57	
Women	−13.45	−13.52 to −13.39		1.56		

**Age group**			<0.001			<0.001
18–24	Base level			1.00		
25–29	3.45	3.28–3.61		1.02	1.01–1.04	
30–34	5.45	5.29–5.62		0.96	0.94–0.97	
35–39	7.65	7.49–7.82		0.84	0.83–0.85	
40–44	9.08	8.92–9.25		0.83	0.82–0.84	
45–49	9.45	9.29–9.61		0.88	0.87–0.89	
50–54	9.30	9.13–9.46		0.97	0.96–0.98	
55–59	8.23	8.07–8.39		1.09	1.08–1.10	
60–64	6.94	6.78–7.09		1.28	1.27–1.30	
65–69	4.85	4.69–5.01		1.57	1.55–1.59	
70–74	2.63	2.47–2.80		1.77	1.75–1.79	
75–79	−0.20	−0.37 to −0.03		1.77	1.75–1.79	
80–84	−3.80	−3.99 to −3.61		1.50	1.48–1.53	
85–89	−7.70	−7.93 to −7.47		1.13	1.11–1.15	
90–94	−10.7	−11.06 to −10.34		0.78	0.76–0.80	
95–99	−14.4	−15.15 to −13.65		0.52	0.50–0.55	

**Townsend score**			<0.001			<0.001
Quintile 1 (least deprived)	Base level			1.00		
Quintile 2	0.48	0.39–0.58		1.08	1.08–1.09	
Quintile 3	0.81	0.71–0.91		1.17	1.17–1.18	
Quintile 4	0.92	0.83–1.02		1.25	1.24–1.26	
Quintile 5 (most deprived)	0.23	0.12–0.34		1.43	1.42–1.44	

**Indicators of diseases**						
Myocardial infarction	−0.19	−0.34 to –0.04	0.015	2.18	2.15–2.21	<0.001
Stroke	−0.75	−0.89 to –0.61	<0.001	1.38	1.37–1.40	<0.001
Diabetes	7.08	7.01–7.15	<0.001	2.53	2.52–2.55	<0.001

**Notes:**

a Differences in the mean weight measurements (in kg) from a multivariable linear regression model, conditional on sex, age group, social deprivation, and indicators of chronic diseases.

b *P*-values from joint Wald tests.

c ORs of having a weight measurement recorded from a multivariable logistic regression model, conditional on sex, age group, social deprivation, and indicators of chronic diseases.
